# Characterization of a *Plasmodium berghei* sexual stage antigen PbPH as a new candidate for malaria transmission-blocking vaccine

**DOI:** 10.1186/s13071-016-1459-8

**Published:** 2016-04-02

**Authors:** Xu Kou, Wenqi Zheng, Feng Du, Fei Liu, Meilian Wang, Qi Fan, Liwang Cui, Enjie Luo, Yaming Cao

**Affiliations:** Department of Pathogen Biology, College of Basic Medical Sciences, China Medical University, Shenyang, Liaoning 110001 China; College of Animal Husbandry and Veterinary, Liaoning Medical University, Jinzhou, Liaoning 121001 China; Department of Immunology, College of Basic Medical Sciences, China Medical University, Shenyang, Liaoning 110001 China; Dalian Institute of Biotechnology, Dalian, Liaoning China; Department of Entomology, The Pennsylvania State University, University Park, PA 16802 USA

**Keywords:** *Plasmodium berghei*, Malaria, PH domain protein, Sexual development, Gametocyte, Ookinete, Transmission-blocking vaccine

## Abstract

**Background:**

Transmission-blocking vaccines (TBVs) are a promising strategy for malaria control and elimination. However, candidate TBV antigens are currently limited, highlighting the urgency of identifying new antigens for TBV development.

**Methods:**

Using a combination of bioinformatic analysis and functional studies in the rodent malaria model *Plasmodium berghei*, we identified a conserved *Plasmodium* protein PbPH (PBANKA_041720) containing a pleckstrin homology (PH) domain. The expression of PbPH was detected by Western blot and indirect immunofluorescence assay (IFA). The function of PbPH was tested by genetic knockout. The TB activity was confirmed by in vitro ookinete conversion assay and mosquito feeding.

**Results:**

PbPH was detected in Western blot as highly expressed in sexual stages (gametocytes and ookinetes). IFA revealed localizations of PbPH on the surface of gametes, zygotes, and ookinetes. Deletion of the *pbph* gene did not affect asexual growth, but significantly reduced the formation of gametocytes, ookinetes, and oocysts, indicating that PbPH protein is required for parasite sexual development. Recombinant PbPH expressed and purified from bacteria elicited strong antibody responses in mice and the antibodies significantly inhibited exflagellation of male gametocytes and formation of ookinetes in a concentration-dependent manner. Mosquito feeding experiments confirmed that mosquitoes fed on mice immunized with PbPH had 13 % reduction in the prevalence of infection and almost 48 % reduction in oocyst density.

**Conclusions:**

*Pbph* is a highly conserved *Plasmodium* gene and is required for parasite sexual development. PbPH protein is expressed on the surface of gametes and ookinetes. Immunization of mice against the recombinant PbPH protein induced strong antibody responses that effectively reduced the formation of male gametes and ookinetes in vitro and blocked transmission of the parasites to mosquitoes. These results highlight PbPH as a potential TBV candidate that is worth future investigations in human malaria parasites.

**Electronic supplementary material:**

The online version of this article (doi:10.1186/s13071-016-1459-8) contains supplementary material, which is available to authorized users.

## Background

Malaria, transmitted by *Anopheles* mosquitoes, is one of the world’s most challenging public health problems. While insecticide-treated nets, antimalarial drugs, and indoor residual sprays of insecticides have together contributed to a significant decrease in the incidence of malaria in many parts of world [1], the emergence and spread of drug-resistant parasites and insecticide-resistant mosquitoes are ever-present risks that potentially threaten the recent gains in malaria control. Interruption of malaria transmission from host to mosquito has been recognized as one of the greatest challenges in malaria elimination [2]. Novel tools that specifically reduce the transmission of malaria parasites from humans to mosquitoes are urgently needed for this purpose. Transmission-blocking vaccines (TBVs) targeting sexual and/or sporogonic development of the parasite and designed to prevent malaria transmission in endemic regions are a potentially highly effective strategy especially during malaria elimination [3].

TBVs are aimed at blocking malaria transmission by interrupting the parasite’s life - cycle in the mosquito. The fundamental principle of TBVs is immunization of humans with surface antigens of sexual- and mosquito-stage parasites to produce antibodies that arrest subsequent development of the parasite in the mosquito midgut, thus cutting off the transmission of malaria parasites [4]. Alternatively, TBV may target mosquito antigens that are required for successful development of the parasite in its vector [5]. TBVs do not directly protect vaccinated individuals from the disease but, rather, protect communities from the spread of malaria. Careful selections of candidate antigens are essential for the development of TBVs. For parasite antigens, the TBV candidates should be localized on the surface of sexual- and mosquito-stage parasites (i.e. gametocyte, gamete, zygote, and ookinete) [6, 7]. To date, several TBV targets have been investigated, and they have distinct characteristics [8, 9]. Antibodies raised against Pfs230 can prevent oocyst development and also lyse gametes in a complement-dependent manner [10]. Antibodies against the male gamete antigen P48/45 are found in human sera from endemic areas and correlate with transmission blocking (TB) activities [11]. Antisera against native or heterologously expressed major ookinete surface antigens P25 or P28 completely inhibit parasite development in mosquitoes [12]. However, most of the existing TBV candidates have been found to be suboptimal [13], and therefore, there is a real need for TBV antigen discovery.

In this study, we mined the *Plasmodium* database and identified a highly conserved *Plasmodium* gene referred to as PbPH, which encodes a hypothetical protein expressed in sexual stages. We confirmed the expression of this protein in *P. berghei* sexual stages and localized it on the surface of gametes and ookinetes. We further evaluated its functions during sexual development by genetic knockout. Immunization of mice against recombinant PbPH protein induced strong antibody responses that effectively blocked transmission of the parasites to mosquitoes.

## Methods

### Bioinformatics

The *pbph* genomic sequences used in this study were retrieved from PlasmoDB, (http://www.plasmodb.org). Putative signal peptide and functional domains were predicated using the SMART online server (http://smart.embl-heidelberg.de/). The presence of a potential GPI anchor in this protein was predicted using PredGPI (http://gpcr2.biocomp.unibo.it/gpipe/). Multiple sequence alignments were performed using the ClustalW multiple sequence alignment program.

### Mice, parasites and mosquitoes

This study used six, eight-week old female BALB/c mice and the *Plasmodium berghei* (ANKA strain 2.34). Mice were purchased from Beijing Animal Institute (Beijing, China). *Anopheles stephensi* (Hor strain) mosquitoes were reared at 25 °C, 50–80 % humidity using a 12 h light dark cycle in an insectary, and fed a 10 % (w/v) glucose solution.

### Ethical approval

Animal use was carried out following the guidelines of the animal ethics committee of China Medical University.

### Cloning, expression and purification of PbPH

*P. berghei* RNA was extracted from *P. berghei*-infected mouse blood 4 days after infection, and cDNA was synthesized using an RNA-to-cDNA kit (Takara). The *pbph* gene was amplified with primers *pbph*-F and *pbph*-R (Additional file 1, Table S1) using *P. berghei* cDNA as the template. The PCR fragment was cloned into the expression vector pET30a (+) at the *Nde*I and *Hin*dIII sites and transformed in *Escherichia coli* BL-21. Protein expression was induced at 20 °C for 12 h in the presence of isopropyl-β-D-thiogalactoside (Promega) at a final concentration of 1 mM and 1 % anhydrous D-glucose. Bacterial cells were lysed on ice by sonication for 15 cycles (20 s pulses with 30 s intervals between each cycle) and the resultant suspension passed through a 0.22-μm filter. The filtered supernatant was loaded onto a Ni-NTA His•Bind Superflow column (Millipore) and purified according to the manufacturer’s protocol. Purified protein was dialyzed overnight in phosphate buffered saline (PBS, pH 7.2) containing 0.2 mM PMSF at 4 °C and an aliquot was analyzed on a 12 % SDS-PAGE gel. Protein samples were quantified using a Bradford assay.

### Immunization and anti-PbPH serum

Six, eight-week old female BALB/c mice (*n* = 6) were immunized by subcutaneous injection of 40 μg recombinant protein emulsified in complete Freund’s adjuvant on day 0 [14], followed by two booster injections (at two-week intervals) with the same amount of protein, respectively. Control mice (*n* = 6) were injected with PBS using the same immunization procedure. Before each immunization and 10 days after the last immunization, blood was collected from the tail vein of each mouse and allowed to clot at room temperature.

Antiserum titers against PbPH were analyzed by ELISA as previously described [15]. A 96-well plate was coated with the recombinant PbPH protein at 10 μg/mL overnight at 4 °C, and then blocked with 5 % skim milk in 1 × PBS, 0.05 % Tween 20 (PBS-T) for 2 h at room temperature. Mouse antiserum was diluted in PBS containing 10 % calf serum albumin (pH 7.2), added to the wells, and incubated for 2 h at 37 °C. After two washes with TBS-T, HRP-conjugated goat anti-mouse IgG antibodies (Invitrogen) diluted 1:5000 was added and incubated for 2 h at 37 °C. After five washes with PBS-T, the chromogenic substrate was added and developed for 5 min. The reaction was stopped by adding 50 μL of 2 mM H_2_SO_4_ to each well. The plate was immediately read with a plate reader at 490 nm.

### Parasite collection, lysate preparation and Western blot

For the collection of schizonts, female BALB/c mice were injected intraperitoneally (i.p.) with 0.2 mL of 6 mg/mL phenylhydrazine in 0.9 % NaCl, 3 days prior to infection. Infection was initiated by injection of 200 μl of mouse blood containing 1 × 10^6^*P. berghei*-infected red blood cells (iRBCs). Four days post-infection, when the parasitemia reached approximately 1 %, mice were anesthetized and heparinized blood collected in a 50 mL tube containing 20 mL of blood-stage culture medium (RPMI 1640, 50 mg/L penicillin, 50 mg/L streptomycin, 100 mg/L neomycin, 20 % [v/v] heat-inactivated fetal calf serum [FCS]). The blood and culture medium were mixed and placed in flasks then incubated in 10 % O_2_, 5 % CO_2_ and 85 % N_2_ at 37 °C, overnight [16]. The culture was then fractionated on a 55 % (v/v) Nycodenz culture gradient medium. To collect gametocytes, mice were treated with sulfadiazine (20 mg/L in drinking water) four days post-*P. berghei* infection for two days to eliminate asexual stage parasites. Six days post-infection, parasites were harvested and kept on ice to avoid premature activation and then separated on a 48 % (v/v) Nycodenz in RPMI 1640 culture medium gradient [17]. Gametocytes were harvested from the interface and washed twice in RPMI 1640. For ookinete collection, mice were bled by cardiac puncture under terminal anesthesia three days post-*P. berghei* infection. Blood was passed through a CF11 cellulose (Whatman) column pre-equilibrated with complete ookinete culture medium (RPMI 1640, 50 mg/L penicillin, 50 mg/L streptomycin, 100 mg/L neomycin, 20 % [v/v] heat-inactivated FCS, 6 U/mL heparin, pH 8.3). The eluate was diluted 1:10 with complete ookinete medium into a wide flask to a maximum depth of 1 cm and kept at 19 °C for 24 h [18]. Parasites were then purified on a 62 % (v/v) Nycodenz/ookinete culture medium gradient.

After collection, parasite pellets were washed twice with PBS containing protease inhibitors (PBS-PI) and proteins were extracted with PBS-PI containing 2 % SDS for 30 min at room temperature [19]. Equal amounts of parasite antigens (10 μg) were electrophoresed on a 12 % SDS-PAGE gel and transferred to a 0.22-μm PVDF membrane (Bio-Rad). The membrane was incubated with mouse anti-PbPH antiserum at 1:200 in Tris-buffered saline containing 0.1 % Tween 20 (TBST) for 3 h at room temperature, washed three times in TBST, and then incubated for 2 h at 37 °C with HRP-conjugated goat anti-mouse IgG antibodies (Invitrogen) diluted at 1:5,000 in TBST. Proteins on the blot were visualized with a Pierce ECL Western Blotting Kit (Thermo Scientific).

### Indirect immunofluorescence assay (IFA)

*P. berghei* asexual stages, gametocytes and subsequent developmental stages (gametes, zygotes and ookinetes) were fixed with 4 % paraformaldehyde in PBS for 15 min at room temperature, rinsed with 50 mM glycine in PBS, and blocked with PBS containing 5 % skimmed milk for 60 min at 37 °C. Slides were incubated with mouse anti-PbPH sera (1:200) or anti-Pbs21 mAb (clone 13.1, 1:500) at 37 °C for 60 min, washed 3 times in PBS, and then incubated with FITC-labeled goat anti-mouse IgG (1:500, Invitrogen) at 37 °C for 30 min. Parasite nuclei were counter-stained with 1 μg/mL of 4′, 6-diamidino-2-phenylindole (DAPI; Invitrogen) [20]. Slides were then mounted with ProLong® Gold anti-fade reagent (Invitrogen) and visualized by fluorescence microscopy. Images were captured and processed on a Zeiss Axio Observer Z2 using Axiovision software and Adobe Photoshop.

### Generation of *pbph* knockout parasites

The *pbph* gene was knocked out using the double-crossover homologous recombination technology as previously described [21]*.* A 777-bp upstream region was amplified by PCR from *P. berghei* genomic DNA using KOD Plus DNA polymerase (Toyobo) with primers 5UTR-F and 5UTR-R (Additional file 1, Table S1), while the 772-bp downstream fragment was amplified with primers 3UTR-F and 3UTR-R (Additional file 1, Table S1). Subsequently, the 777-bp fragment was digested by *Hin*dIII and *Pst*I and the 772-bp fragment were digested by *Xho*I and *Eco*RI. The digested PCR products were inserted into the PL0034 plasmid to flank the *hdhfr* expression cassette. The plasmid (10 μg) was linearized with *Xho*I and electroporated into cultured *P. berghei* schizonts using the Nucleofector II. Transfected parasites were delivered into female BALB/c mice by intravenous injection and mice were treated with pyrimethamine (70 μg/mL) 24 h later *via* drinking water. Infected blood was collected to examine the integration of the disruption cassette by PCR using integration-specific primers (Additional file 1, Table S1). Parasites were cloned by limiting dilution.

### Phenotypic analysis of *Δpbph* parasites during asexual and sexual stages

To study the functions of PbPH during *Plasmodium* sexual development, ten mice were pre-treated with phenylhydrazine. Five mice each were injected with 1 × 10^6^*Δpbph*-iRBCs, while the other five were injected with 1 × 10^6^ wild type *P. berghei-*iRBCs*.* Parasitemia was monitored daily by Giemsa-stained blood smears. On day 3 after infection, gametocytemia (mature gametocytes per 100 RBCs) and gametocyte sex ratio were determined by Giemsa-stained, tail blood smears [22]. Exflagellation of male gametocytes was quantified as described [23]. Briefly, 10 μl of gametocyte-infected blood was obtained from the tail vein and mixed immediately with 90 μl of complete ookinete culture medium. The mixture was placed under a Vaseline-coated coverslip at 25 °C, and 15 min later exflagellation centers were counted over the next 10 min under a phase contrast microscope. Similarly, ookinete formation was determined using 10 μl of blood collected from the tail vein of each mouse, which was drawn into 90 μl ookinete culture medium and incubated at 19 °C for 24 h. Cultured ookinetes were labeled with a mouse anti-Pbs21 antibody (1:500) and enumerated under a fluorescence microscope.

For mosquito feeding experiment, starved *A. stephensi* mosquitoes (~50/mouse) were allowed to feed on the infected mice at three days post-infection for 30 min [24, 25]. Unfed mosquitoes were removed and fed mosquitoes were maintained at 19–22 °C and in 50–80 % relative humidity. For each mouse, up to 30 mosquitoes were dissected 10 days after feeding. The midguts were stained with 0.5 % mercurochrome (Sigma-Aldrich). Oocysts were counted to determine the prevalence of infection (number of infected mosquitoes) and the intensity of infection (number of oocysts per positive midgut).

### Quantification of TB activity

To determine whether immune sera against PbPH affect exflagellation of male gametocytes, 10 μl of infected mouse blood was mixed with the ookinete culture medium containing anti-PbPH mouse sera or control sera at final dilutions of 1:5, 1:10 and 1:50. Quantification of exflagellation centers was performed as described above.

For the *in vitro* ookinete conversion assay, mice were treated with phenylhydrazine and inoculated i.p. with 0.2 ml of infected blood containing 1 × 10^6^*P. berghei* parasites*.* Blood was collected from mice three days after infection. After removal of the serum, blood was reconstituted using control sera or sera from mice immunized with the recombinant PbPH protein at 1:5, 1:10, and 1:50 dilution with the complete ookinete culture medium [26, 27]. Parasites were cultured *in vitro* at 19 °C for 24 h. The cultures were fixed, labeled with Pbs21 mAb (1:500), and ookinete numbers quantified under a fluorescence microscope. Sera from female BALB/c mice injected with PBS were used as negative controls.

For mosquito feeding experiment, two groups of mice (*n* = 5) were immunized with recombinant PbPH protein or PBS (negative control) as described above. Seven days after the final boost, all mice were treated with phenylhydrazine, and infected i.p. with 1 × 10^6^*P. berghei* iRBC. Mosquito feeding and oocyst counting were performed as described above.

### Statistical analyses

Statistical comparison between groups (IgG levels, parasitemia, gametocytemia, ookinete numbers) was made by Student’s *t* test using the GraphPad Prism software. The intensity of infection (oocysts/midgut) was analyzed using Mann–Whitney *U* test, while infection prevalence was analyzed by Fisher’s exact test using SPSS version 17.0.

## Results

### PbPH is highly conserved among *Plasmodium* species

**Fig. 1:**
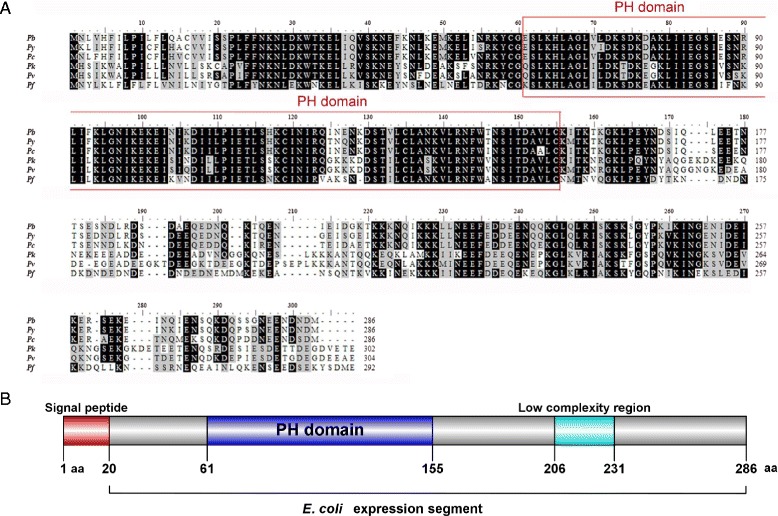
Bioinformatic analyses of PbPH. **a** Alignment of protein sequences of PH domain proteins in *Plasmodium* species:  *P. berghei* (*Pb*), *P. falciparum* (*Pf*), *P. yoelii* (*Py*), *P. chabaudi* (*Pc*), *P. vivax* (*Pv*), and *P. knowlesi* (*Pk*). Amino acids conserved across six species are marked: identical (black), similar (gray). The PH domain is highlighted. **b** Schematic diagram of PbPH showing the locations of a signal peptide (red) and a PH domain (blue). The *E. coli* expression segment shows amino acids 21 to 286

Through bioinformatic analysis using criteria of protein expression in sexual stages and the presence of a putative signal peptide, we identified a *P. berghei* gene PBANKA_041720 from the *Plasmodium* database. This gene is located on chromosome 4, encoding a protein of 286 amino acids with a calculated molecular weight of 33 kDa. The predicted protein contains a signal peptide and a pleckstrin homology (PH) domain (Fig. 1b), and therefore, it is referred to as PbPH. PH domains are small modular domains that occur in a large variety of eukaryotic signaling proteins. The domains can bind to phosphatidylinositol in biological membranes and also to proteins such as protein kinase C [28]. Through such interactions, PH domains recruit proteins to different membranes and target them to appropriate cellular compartments or enable them to interact with other components of a signal transduction pathway [27, 29, 30]. PredGPI did not predict the presence of a GPI anchor within PbPH. Multiple sequence alignments using a ClustlW showed that this PH domain protein is highly conserved among *Plasmodium* species and PH domain is the most homologous sequence (Fig. 1a).

## PbPH is expressed in sexual stages

**Fig. 2:**
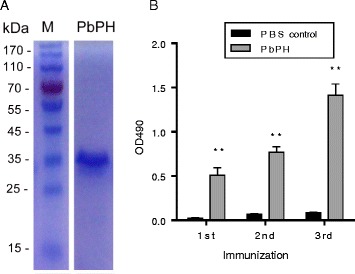
Recombinant PbPH protein purification and immunization. **a** Purified recombinant proteins were subjected to electrophoresis on a 12 % SDS-PAGE gel. M: PageRuler Prestained Protein Ladder in kDa. **b** ELISA showing the PbPH-antibody titers after immunizations. Serum samples were collected post-immunization on days 14, 28, and 38. Titers correspond to the last dilution of the test serum in which OD490 values were above that of the cut-off. The cut-off value was defined as that of pooled sera from native mice. Serum samples were tested at two-fold serial dilutions (1 × 10^2^–2 × 10^5^). Pooled sera from native mice were used as a negative control. Results are representative of three independent experiments. The error bars indicate mean ± SEM (*n* = 3). **P < 0.01 (Student’s *t* test)

The recombinant PbPH protein was expressed in *E. coli* as His-tagged protein and purified by affinity chromatography using Ni-NTA resin. SDS-PAGE analysis revealed that the recombinant PbPH protein had a molecular weight of approximately 33 kDa, consistent with the predicted molecular size of PbPH (Fig. 2a). The recombinant PbPH was used to immunize mice to produce polyclonal antibodies. ELISA showed that mice developed a strong antibody response against recombinant PbPH. Antibody titers increased over time following the initial immunization, with substantial boosting observed in the two subsequent immunizations. Antibody titers were significantly higher than those from PBS control (*t*_(4)_ =10.21, *P* = 0.0005; Fig. 2b) and it reached a titer of 1:25600 after the third immunization. This result indicated that the recombinant PbPH was highly immunogenic and successfully induced the production of specific antibodies in mice.

**Fig. 3:**
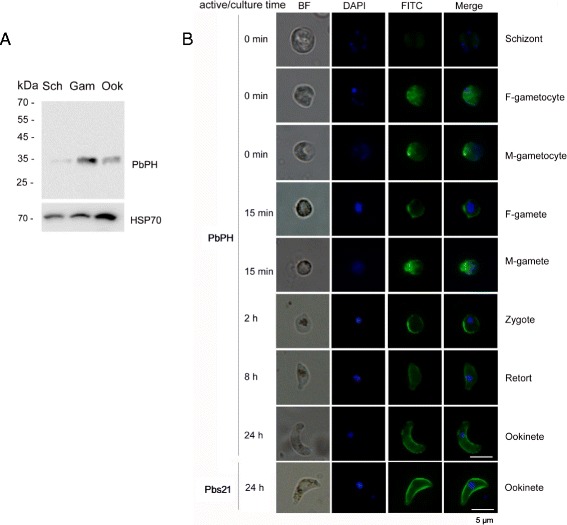
Expression and localization of PbPH in the parasites. **a** Western blot of PbPH in asexual- and sexual-stage parasites. Parasite antigens of schizonts, gametocytes, and ookinetes (10 μg/lane) were incubated with anti-PbPH sera (1:200) from immunized mice. Protein loading is estimated using anti-Hsp70 serum (1:200). **b** IFA analysis. Blood smears containing parasite samples were stained at different times after collection of *P. berghei*-infected blood. Parasites were fixed and stained with anti-PbPH sera (1:200) from recombinant PbPH-immunized mice and then with FITC-conjugated goat anti-mouse IgG (green). Nuclei were stained by DAPI (blue). Pbs21 mAb clone 13.1 serves as a positive control. Scale - bar: 5 μm

To determine whether these antibodies are reactive with the native PbPH protein in *P. berghei*, Western blot was performed with parasite lysates from purified schizonts, gametocytes and ookinetes. The anti-PbPH antisera primarily recognized a band at approximately 33 kDa in purified gametocytes and ookinetes (Fig. 3a). Only a very faint band was detected in schizonts, which may be due to the small number of contaminating gametocytes. This is consistent with the published *P. berghei* proteome data showing that the PbPH protein is expressed in gametocytes and ookinetes [31, 32]. This result also confirmed that the anti-PbPH sera recognized the native parasite antigen.

The localization of PbPH in the asexual and sexual stages of *P. berghei* was investigated by IFA. Whereas no signal was detected in schizonts, strong fluorescence was observed in male and female gametocytes, and on the surfaces of male and female gametes, zygotes, retorts and ookinetes (Fig. 3b). Also consistent with the Western blot, strongest fluorescence was observed in gametocytes and gametes. Compared with the surface location of Pbs21, the PbPH localization on the ookinete surface was less homogeneous. Surface localization is consistent with the presence of a signal peptide in PbPH and indicates that this protein is secreted in sexual stages.

### PbPH is needed for sexual development in *P. berghei*

**Fig. 4:**
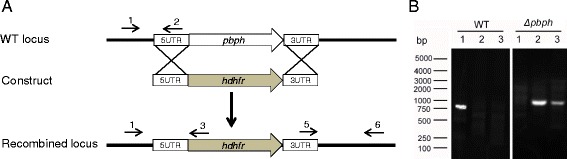
Knockout of *pbph* gene from *P. berghei* parasite. **a** A schematic shows the wild type *pbph* locus, transfection construct, and the recombined locus. Through a double-crossover strategy the pbph locus was replaced with the *dhfr*-expressing cassette. Primers 1–6 used to detect gene knockout are marked. **b** PCR detection of wild-type and *Δpbph* parasite genomes. Lane 1: primers 1 + 2 (910 bp); Lane 2: primer 1 + 3 (990 bp); and Lane 3: primer 5 + 6 (1, 040 bp)

**Fig. 5:**
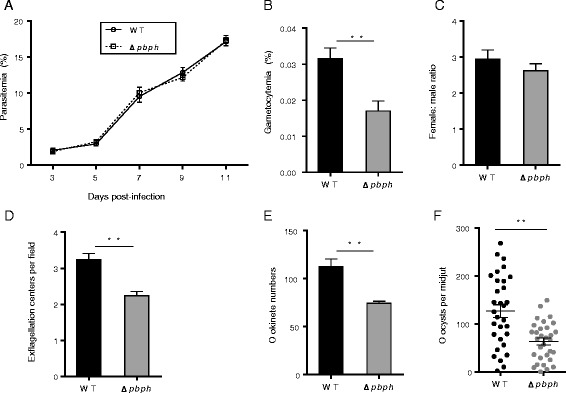
Functional analysis of PbPH during parasite development. **a** Mice were infected with *P. berghei* or *Δpbph* parasite and parasitemia was monitored for 11 days. **b** Gametocytemias in mice infected with wild-type (WT) and *Δpbph* parasites. **P < 0.01 (Mann–Whitney *U* test). **c** Female: male gametocyte ratios of WT and *Δpbph* parasites. **d** Exflagellation of male gametocytes in WT and *Δpbph* parasites. **P < 0.01 (Student’s *t* test). **e** Ookinete numbers in WT and *Δpbph* parasites. **P < 0.01 (Student’s *t* test). The error bars in B-E indicate mean ± SEM (*n* = 5). **f** Oocyst numbers per midgut in mosquitoes fed on WT and *Δpbph* parasites. **P < 0.01 (Mann–Whitney *U* test). The horizontal bars indicate the mean number of oocysts per midgut (± SEM). All the data in the functional analysis of PbPH are representative of three independent experiments

To study the functions of PbPH in parasite development, the *pbph* gene was knocked out by homologous recombination (Fig. 4a). *Pbph* knockout-lines (*Δpbph*) were selected by pyrimethamine and cloned by limiting dilution. After cloning, deletion of the *pbph* gene was confirmed by integration-specific PCR in three clones (Fig. 4b). Phenotypic comparison between the wild-type and *Δpbph* parasite was performed in both asexual and sexual stages. Consistent with the lack of *pbph* expression in asexual erythrocytic stages, deletion of the *pbph* gene in *P. berghei* did not result in a noticeable difference in parasitemia as compared with the wild-type parasite (Fig. 5a), indicating that PbPH is dispensable during the asexual stages of the parasites. However, compared to the wild type, the *Δpbph* line displayed a significantly reduced production of gametocytes (*t*_(8)_ = 3.63, *P* = 0.0067; Fig. 5b). Gametocytemia had a 1.8 fold reduction in the *Δpbph* parasites compared with the wild type. Deletion of *pbph* appeared to have affected the formation of both male and female gametocytes, since there was no significant difference in sex ratio between the wild-type and *Δpbph* parasites (Fig. 5c). To determine the function of *pbph* in subsequent sexual development, we quantified exflagellation and ookinete conversion from the same numbers of gametocytes. The results showed that the *Δpbph* line displayed a ~31 % reduction in exflagellation centers per field (*t*_(8)_ = 4.81, *P* = 0.0013; Fig. 5d). Ookinete conversion *in vitro* was also severely affected; mature ookinete number had a 34 % reduction in the *Δpbph* line compared to the wild-type (*t*_(8)_ = 4.74, *P* = 0.0015; Fig. 5e). In mosquito feeding assays, the mean number of oocysts per midgut was reduced from 126.9 in those fed on wild-type parasites to 63.7 in those fed on *Δpbph* parasites (*Z* = -3.41, *P* = 0.001; Fig. 5f). It is noteworthy that the declines in ookinetes and oocysts could be the result of reduced gametocyte and gamete numbers.

## Antibodies against PbPH can block parasite sexual development

**Fig. 6:**
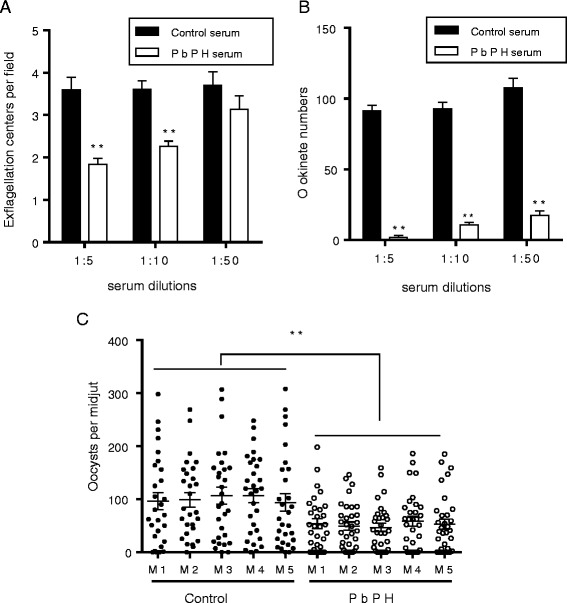
Transmission-blocking activity of PbPH. **a** Effect of anti-PbPH sera on exflagellation of male gametocytes. Exflagellation centers per field were measured after 15 min incubation with anti-PbPH mice sera or control sera at final dilutions of 1:5, 1:10 and 1:50. Means were calculated from three separate experiments. Error bars indicate mean ± SEM (*n* = 3). **P < 0.01 (Student’s *t* test). **b** The effect of anti-PbPH sera at 1:5, 1:10, and 1:50 dilutions on *P. berghei* ookinete conversion *in vitro*. Means were calculated from three separate experiments. Error bars indicate mean ± SEM (*n* = 3). *P < 0.05; **P < 0.01 (Student’s *t* test). **c** Direct mosquito feeding assay on control mice and mice immunized with recombinant PbPH protein (5 mice per group). Mice were subsequently infected with wild-type *P. berghei* and used to assess TB activities. The data are representative of three independent experiments. Data points represent midgut oocyst numbers of individual mosquitoes in each group. Mean number of oocysts per midgut and the SEM are shown

To study the TB activities of the antibodies against PbPH, we first evaluated the effect of the antibodies on the formation of male gametes. When wild type *P. berghei* gametocytes were incubated with the anti-PbPH antisera at the 1:5 and 1:10 dilutions, male gametogenesis was significantly affected. Compared to control sera, the number of exflagellation centers per field were reduced by 49 and 37 % when incubated with anti-PbPH serum at 1:5 (*t*_(4)_ = 5.46, *P* = 0.0054; Fig. 6a) and 1:10 (*t*_(4)_ = 5.54, *P* = 0.0051; Fig. 6a) dilutions, respectively.

Given the localization of PbPH on the surfaces of sexual stages, we wanted to further evaluate TB activity of antibodies against PbPH using an *in vitro* ookinete conversion assay and mosquito feeding. In the *in vitro* assay, immune sera against PbPH at all dilutions used exhibited significant effects in blocking the formation of ookinetes (Fig. 6b). In particular, the anti-PbPH sera at 1:5 dilution almost completely blocked ookinete formation (t _(4)_ = 21.40, *P* < 0.0001; Fig. 6b). Even at 1:50 dilution, the ookinete number was reduced by 84 % when cultured with the immune sera (*t *_(4)_ = 12.26, *P* = 0.0002; Fig. 6b).

To further test the TB efficacy of antibodies induced by recombinant PbPH *in vivo*, immunized mice were used to feed *An. stephensi* mosquitoes. Compared to the control groups, mosquitoes fed on recombinant PbPH-immunized mice showed a significantly lower number of oocyst intensity. Whereas the mean oocyst intensity was 100.5 in mosquitoes fed on control mice, it was almost halved (52.3) in mosquitoes fed on PbPH-immunized mice (*Z* = -5.28, *P* < 0.0001; Fig. 6c, Table 1). Further, mosquitoes fed on control mice displayed an average infection prevalence of 93.7 %, whereas it was reduced to 80.6 % in mosquitoes fed on PbPH-immunized mice (OR = 3.571, 95 % CI = 1.631–7.818, *P* < 0.001; Table 1), demonstrating modest TB activity of antibodies against recombinant PbPH.

**Table 1:** Mosquito infection prevalence and oocyst intensity after feeding on control mice and mice immunized with recombinant PbPH

	Control mice ^a^					PbPH-immunized mice ^a^				
	M1	M2	M3	M4	M5	M1	M2	M3	M4	M5
Mosquitoes Infected/Dissected	28/25	28/27	29/27	30/29	29/27	30/24	32/27	31/25	30/24	32/25
Prevalence of infection (%) ^b^	89.3	96.4	93.1	96.7	93.1	80	84.4	80.6	80	78.1
Mean prevalence (%)					93.72					80.62
Reduction in prevalence (%)^c^										13.1
Oocyst intensity ^d^	96.5	98.70	106.80	106.60	93.97	54.10	49.53	46.65	58.39	52.94
SEM ^e^	16.04	13.68	16.08	16.08	16.78	9.20	7.66	7.74	9.54	9.68
Mean oocyst intensity					100.51					52.32
Reduction in oocyst intensity ^f^										47.95

^a^ Five mice were used in each group for the evaluation of TB activity

^b^ The prevalence of infection was calculated as the number of mosquitoes with oocysts/total mosquitoes dissected in each group × 100%

^c^ The percent reduction of prevalence was calculated as % mean prevalence _control_ – % mean prevalence _PbPH_

^d^ Mean number of oocysts per mosquito midgut

^e^ Standard error of the mean

^f^ The percent reduction in oocyst intensity was calculated as (mean oocyst intensity_control_ – mean oocyst intensity_PbPH_)/mean oocyst intensity _control_ × 100%

## Discussion

Interrupting the development of a parasite inside its mosquito vector represents a key component of malaria control strategies [33]. By mining the PlasmoDB, we identified a gene PbPH that is expressed in sexual stages and characterized its functions in sexual development. The surface localization of the PbPH protein on sexual stages prompted us to evaluate its TB activities. Antisera against recombinant PbPH were found to reduce the formation of male gametes, block the formation of ookinetes *in vitro*, and reduce the infection prevalence and oocyst density in mosquito feeding experiments.

PbPH is a conserved membrane protein of *Plasmodium* expressed in gametocytes, gametes, zygotes, and ookinetes. Whereas the detection of expressed PbPH outside of the cells is consistent with the presence of a signal peptide, the lack of a putative GPI-anchor suggests that its association with the membranes may be through interactions with the membrane directly or with other membrane proteins. The fact that the PH domains can bind phosphatidylinositol in biological membranes [28] suggested that the surface localizations of PbPH was probably achieved through direct binding of the PbPH to phosphatidylinositol. PH domains are commonly found in eukaryotic signal proteins. For example, the calcium-dependent protein kinase PfCDPK7 from *P. falciparum* interacts with phosphatidylinositol phosphate *via* its PH domain, which may guide its subcellular localization [34]. While how PbPH functions during *Plasmodium* sexual development requires further investigations, gene disruption study demonstrated a role of PbPH in gametocyte and gamete development, and possibly subsequent maturation of ookinetes and their invasion of the midgut epithelium.

Surface localization of the target during the sexual stages of *Plasmodium* development is considered a key parameter for the triage and selection of novel TBV candidates [6]. The expression of PbPH on the surface of sexual stages makes it a potential candidate for TBV development. Its expression from gametocytes to ookinetes suggests that antibodies against this protein may interrupt parasite transmission at multiple steps. First, the anti-PbPH antibodies may impede gametocyte and gamete development. Consistently, the formation of male gametes was significantly reduced during *in vitro* incubation of gametocytes with the anti-PhPH antisera. In addition, with the surface localization on both male and female gametes, the anti-PbPH antiserum may interfere with the interactions between the two sexes and effectively prevent mating and formation of zygotes [35]. Furthermore, PbPH was also expressed on the surface of zygotes and ookinetes, suggesting that subsequent transition and maturation of ookinetes may also be affected. In reflecting this possibility, the number of ookinetes was drastically reduced in an *in vitro* ookinete conversion assay. Finally, the presence of PbPH on the surface of ookinetes, albeit at a much reduced level, suggests that antibodies against PbPH may also inhibit the invasion of the midgut epithelium in the mosquitoes. Altogether, these are translated into reduced prevalence of infected mosquitoes and oocyst density in mosquito feeding assays.

Collectively, PbPH could be potential TBV candidate because of its expression in both pre- and post-fertilization stages. Since pre-fertilization antigens are targets of the natural immune responses, immunization of the vertebrate hosts with such antigens may also help boost naturally-occurring antibodies. This study demonstrated evident TB activity of PbPH antibodies for multiple developmental steps, which cooperatively lead to significant reduction in mosquito infectivity. The high levels of conservation of this protein in *Plasmodium* highlight its potential as a target for TBV development and warrants parallel studies in human malaria parasites.

## Conclusions

In this study, we showed that pbph is a highly conserved *Plasmodium* gene and PbPH protein localized it on the surface of gametes, zygotes, retorts and ookinetes. Functional studies of PbPH revealed that knockout of the pbph gene can affect the formation of gametocytes, gametes, ookinetes and oocysts. Anti-PbPH serum significantly reduced the formation of male gametes and ookinetes *in vitro*. Mosquitoes that fed on recombinant PbPH protein-immunized mice showed significantly lower oocyst intensity and infection prevalence compared to that of a control group. In summary, these pivotal findings indicate that PbPH in *Plasmodium* is essential for parasite transmission *via* mosquitoes and this thus highlights its potential as a target for the development of a transmission-blocking vaccine.

## Additional file

Additional file 1: Supplementary Table S1. Primer information. (DOCX 14.2 kb)
