# Erratum to: Characterization of a *Plasmodium berghei* sexual stage antigen PbPH as a new candidate for malaria transmission-blocking vaccine

**DOI:** 10.1186/s13071-017-2021-z

**Published:** 2017-02-16

**Authors:** Xu Kou, Wenqi Zheng, Feng Du, Fei Liu, Meilian Wang, Qi Fan, Liwang Cui, Enjie Luo, Yaming Cao

**Affiliations:** 1 Department of Pathogen Biology, College of Basic Medical Sciences, China Medical University, Shenyang, Liaoning 110001 China; 2 College of Animal Husbandry and Veterinary, Liaoning Medical University, Jinzhou, Liaoning 121001 China; 3 Department of Immunology, College of Basic Medical Sciences, China Medical University, Shenyang, Liaoning 110001 China; 4 Dalian Institute of Biotechnology, Dalian, Liaoning China; 5 Department of Entomology, The Pennsylvania State University, University Park, PA 16802 USA

## Erratum

After the publication of our paper [1], we realized that the positions of the primers 2, 5 and 6 in Fig. 4a were incorrect due to shifting. The corrected Fig. 4 is included below. In addition, “Mosquitoes Infected/Dissected” in Table 1 should read “Mosquitoes Dissected/Infected”. We would like to apologise for these errors and for any inconvenience this may have caused.

**Fig. 4:**
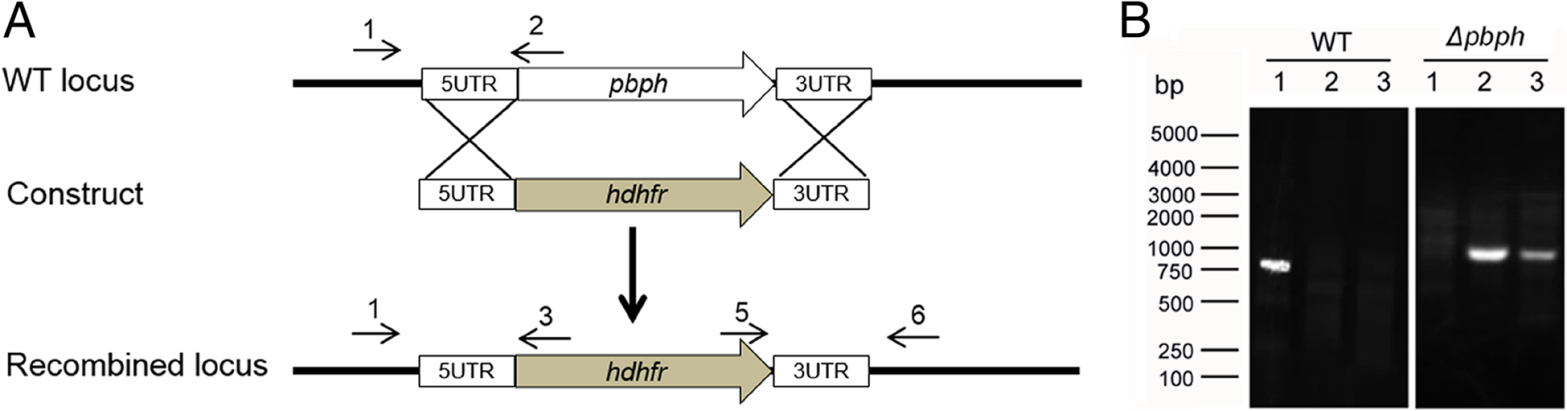
Knockout of *pbph* gene from *P. berghei* parasite. **a** A schematic shows the wild type *pbph* locus, transfection construct, and the recombined locus. Through a double-crossover strategy the *pbph* locus was replaced with the *dhfr*-expressing cassette. Primers 1–6 used to detect gene knockout are marked. **b** PCR detection of wild-type and *Δpbph* parasite genomes. Lane 1: primers 1 + 2 (910 bp); Lane 2: primers 1 + 3 (990 bp); Lane 3: primers 5 + 6 (1,040 bp)
