# Supplementary material for: Characterization of a Plasmodium berghei sexual stage antigen PbPH as a new candidate for malaria transmission-blocking vaccine
Source: Parasit Vectors. 2016 Apr 2;9:190. doi: 10.1186/s13071-016-1459-8 (PMC4818878; doi:10.1186/s13071-016-1459-8)
Supplement: Additional file 1: — Supplementary Table S1. Primer information. (DOCX 14.2 kb) [file 13071_2016_1459_MOESM1_ESM.docx]

**Additional file 1: Supplementary Table S1.** Primer information

| Purpose | Primer name | Sequence |
| --- | --- | --- |
| *E. coli* expression of PbPH | *pbph*-F | CGCCATATGTCACCCTTATTTTTTAATA |
|  | *pbph* -R | CCCAAGCTTCTACATATCGTTATCGTT |
| *Pbph* KO | 5UTR-F | CCCAAGCTTTAAGGGTGATGATTATGGTA |
|  | 5UTR-R | AACTGCAGTGAATAGTCAATTTGGTGGT |
|  | 3UTR-F | CCGCTCGAGGCTAGAAAGACAG |
|  | 3UTR-R | CCGGAATTCGGCTTATTCATGTTAC |
| Confirmation of *Δpbph* parasites | Primer 1 | TAAGGGTGATGATTATGG |
|  | Primer 2 | ACTAATCCCGCTAAATG |
|  | Primer 3 | GGTGCTTTGAGGGGTGAG |
|  | Primer 5 | TTTTTCCTTCAATTTCGGG |
|  | Primer 6 | GAGAAATACTCGGTAGG |
